# *Theroa zethus* Caterpillars Use Acid Secretion of Anti-Predator Gland to Deactivate Plant Defense

**DOI:** 10.1371/journal.pone.0141924

**Published:** 2015-10-30

**Authors:** David E. Dussourd

**Affiliations:** Department of Biology, University of Central Arkansas, Conway, Arkansas, United States of America; Pennsylvania State University, UNITED STATES

## Abstract

In North America, notodontid caterpillars feed almost exclusively on hardwood trees. One notable exception, *Theroa zethus* feeds instead on herbaceous plants in the Euphorbiaceae protected by laticifers. These elongate canals follow leaf veins and contain latex under pressure; rupture causes the immediate release of sticky poisonous exudate. *T*. *zethus* larvae deactivate the latex defense of poinsettia and other euphorbs by applying acid from their ventral eversible gland, thereby creating furrows in the veins. The acid secretion softens the veins allowing larvae to compress even large veins with their mandibles and to disrupt laticifers internally often without contacting latex. Acid secretion collected from caterpillars and applied to the vein surface sufficed to create a furrow and to reduce latex exudation distal to the furrow where *T*. *zethus* larvae invariably feed. Larvae with their ventral eversible gland blocked were unable to create furrows and suffered reduced growth on poinsettia. The ventral eversible gland in *T*. *zethus* and other notodontids ordinarily serves to deter predators; when threatened, larvae spray acid from the gland orifice located between the mouthparts and first pair of legs. To my knowledge, *T*. *zethus* is the first caterpillar found to use an antipredator gland for disabling plant defenses. The novel combination of acid application and vein constriction allows *T*. *zethus* to exploit its unusual latex-bearing hosts.

## Introduction

Lineages of insect herbivores typically specialize on plants that are taxonomically related or that share similar defensive traits such as the presence of latex canals (laticifers) or the production of a particular class of chemicals [1, 2]. Extreme host shifts to unrelated plants bearing drastically different defenses are uncommon, particularly among specialist herbivores. Nevertheless, striking host shifts have occurred within the Notodontidae, a group of over 4,400 species of caterpillars that in North America feed almost exclusively on hardwood trees [3–5]. An unusual species, *Theroa zethus*, feeds not on long-lived trees with tannin-rich leaves, but on herbaceous members of the Euphorbiaceae that emit profuse latex when damaged. Only a single *Theroa* species has been described [6]: *T*. *zethus* (Druce) occurs in southern Arizona where it feeds on hyssopleaf sandmat, *Chamaesyce hyssopifolia* (L.) Small; fire on the mountain, *Euphorbia cyathophora* Murray, toothed spurge, *E*. *dentata* Michx. (D. Wagner unpub. data.), and *C*. *serpyllifolia*. Euphorb feeding is derived within the Notodontidae and probably evolved from tree feeding (J. Miller pers. comm.). This study examines how *T*. *zethus* utilizes such atypical hosts that emit copious latex exudates, which in the Euphorbiaceae are often toxic and sticky [7–9].

Insects that specialize on latex plants commonly deactivate the laticifers by severing or pinching leaf veins with their mandibles [10], thus disrupting the elongate latex canals that typically follow the vascular bundles [11–12]. Severing veins drains latex from the distal section where the insect invariably feeds. More importantly, the cuts isolate the distal branches of the laticifers, thus preventing bulk flow of latex to the insect’s feeding site [13, 14]. Thus, *Amblycorypha* katydids on *Euphorbia* repeatedly snip the leaf midrib with their mandibles before feeding on the leaf tip beyond the cuts [10]. Likewise, an arctiine caterpillar *Pygarctia roseicapitis* (Neumoegen and Dyar) cuts leaf midribs, petioles or stems of euphorb hosts before feeding distal to the cuts [15]. Similarly, caterpillars of the sphingid *Erinnyis ello* (L.) cut elongate trenches across euphorb leaves or constrict and cut into stems and petioles [10, 16, 17]. Such canal-cutting behaviors are widespread among insect folivores on plants that release exudates from elongate canals, being reported in over 90 insect species in 13 families and 3 orders [10].

Some notodontids species employ similar behaviors on trees that lack secretory canals. The caterpillars use their mandibles to chew a shallow girdle that completely encircles stems and petioles or they cut a furrow in the leaf midrib [18]. Larvae of *Schizura leptinoides* (Grote)(recently transferred to the genus *Oedemasia* by Becker [6]) spend ~5–10% of their time cutting girdles and bathe the exposed vascular tissues within each girdle with saliva from their labial salivary glands [18](D.E. Dussourd, M. Peiffer, G.W. Felton, unpub. data). The saliva presumably functions to block induced plant defensive responses as documented for saliva secreted by feeding noctuid caterpillars on *Nicotiana*, *Medicago*, and *Arabidopsis* [19]. Does *Theroa*, which is classified with *Oedemasia* in the subfamily Heterocampinae [6], likewise circumvent the latex defense of their unusual euphorb hosts by chewing girdles and applying saliva like other notodontids or have they adopted vein-cutting behaviors more typical of insects on latex plants?

Unlike other herbivores on laticiferous plants, *T*. *zethus* larvae have available an unusual anti-predator defense that could potentially be usurped to disarm host defenses.

**Fig 1:**
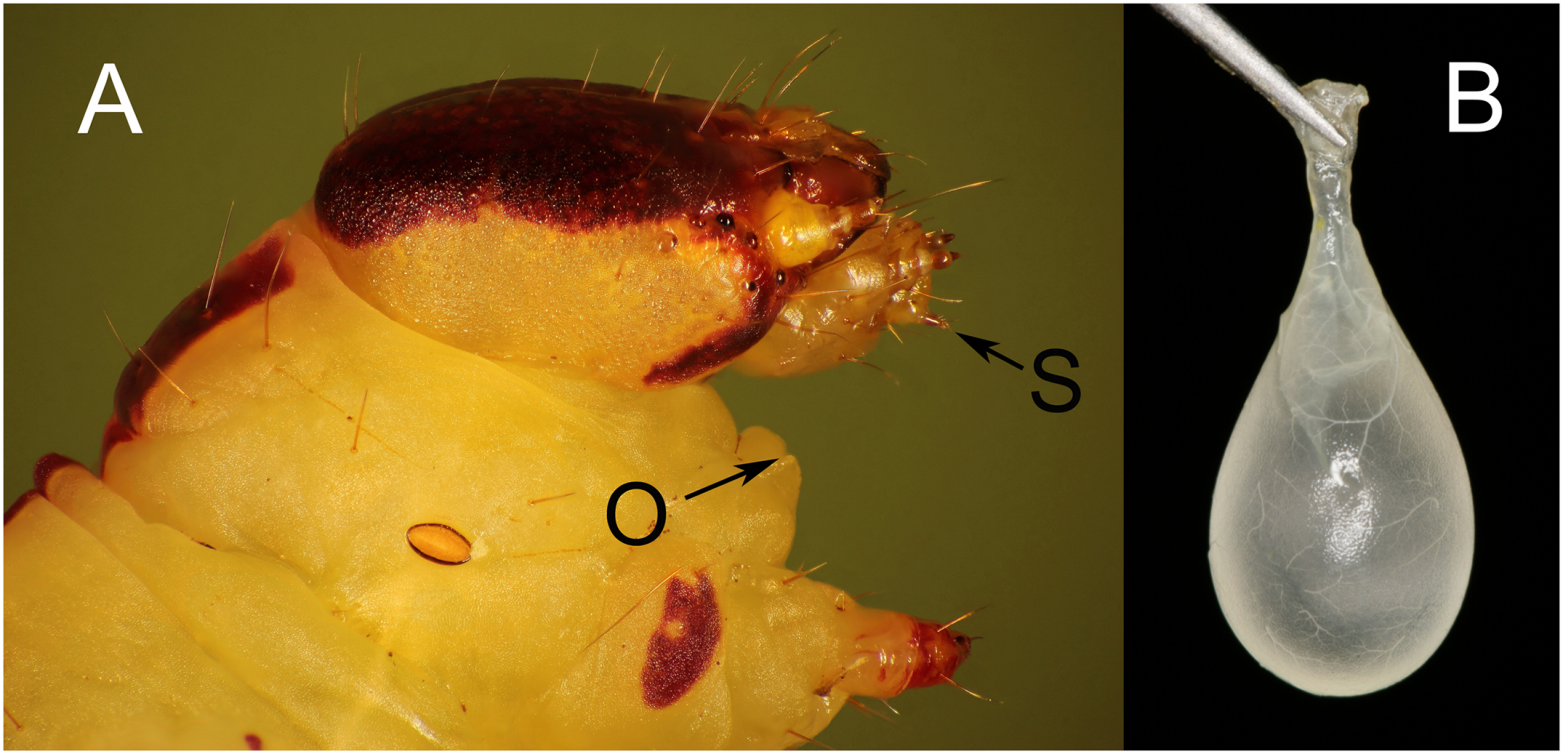
Secretion from the ventral eversible gland (VEG) is released near the mouthparts. (A) Head and prothoracic segment of a final instar *T*. *zethus* showing the location of the orifice (O) for the VEG and of the spinneret (S), which releases saliva from the labial salivary gland. (B) VEG dissected from a final instar *T*. *zethus*.

A large sac-like gland, the ventral eversible gland (VEG) or adenosma, releases acidic secretions from an opening on the ventral surface between the head and first pair of legs (Fig 1). When threatened, notodontids can spray multiple discharges of VEG fluid aimed in any direction (S1 Movie) [20, 21]; various predators including birds, lizards, toads, spiders and ants are reportedly deterred [22, 23]. Chemical analysis documents that notodontid VEG secretions typically contain high concentrations of formic acid, often with smaller amounts of acetic acid and various lipophilic constituents [21, 22, 24–26]. The VEG of *T*. *zethus* likewise contains concentrated formic acid with smaller amounts of butyric acid (K. Rajan, D.J. Carrier, D.E. Dussourd, unpub. data). The location of the VEG opening close to mouthparts suggests that the VEG might be used to circumvent host defenses, although in armyworms it has recently been shown to increase defensive responses from *Arabidopsis* and *Solanum* hosts [27, 28].

This article documents in a series of five experiments that *T*. *zethus* larvae deactivate the latex defense of euphorbs through a combination of vein constriction/cutting and VEG acid application. In the first experiment, larvae from the first to final instar all effectively reduced latex exudation in the euphorb *Chamaesyce maculata* (L.) Small by cutting or compressing veins or stems, behaviors previously noted in other herbivores on latex plants. However, their constrictions often caused necrosis in surrounding tissues, an unexpected consequence not previously reported. The second experiment demonstrated that necrosis is caused by acid secretions from the VEG, not saliva from the labial salivary glands. Poinsettia, *Euphorbia pulcherrima* Willd. Ex Klotzsch, was chosen for this experiment and for subsequent experiments because the leaves have substantial veins that are not easily crimped by mandibles, which facilitated identifying the cause of necrosis. The third experiment showed that *T*. *zethus* larvae apply acid during a unique behavior where they hold the VEG opening pressed tightly against the leaf vein for several minutes (S4 Movie). In the fourth experiment, the VEG secretion by itself sufficed to create withered furrows in poinsettia veins and to reduce distal latex exudation. Finally, the fifth experiment showed that VEG secretions substantially increased growth of *T*. *zethus* on poinsettia. In addition, field observations documented that the behaviors observed on *C*. *maculata* and *E*. *pulcherrima* also occur on recently-discovered native hosts of *T*. *zethus* in Arizona.

## Materials and Methods

### Study organisms

*Theroa zethus* were collected in southern Arizona at the Southwestern Research Station (31°52'57"N and 109°12'14"W) near Portal, Arizona and the Santa Rita Experimental Range and Wildlife Area (31°50'00"N and 110°51'10"W) near Helvetia, Arizona with permission of the station directors and on private land in Ash Canyon (31°29'44"N and 110°51'42"W) near Sierra Vista, Arizona by the homeowner. Females and offspring were imported from Arizona to Arkansas under USDA APHIS permit P526P-12-00188. Newly emerged larvae were reared in the lab on potted *Chamaesyce maculata* (previously *Euphorbia supina*); poinsettia, *Euphorbia pulcherrima* (Eckespoint Classic Red in all experiments), *E*. *cyathophora*, *C*. *hyssopifolia* and other Euphorbiaceae. No endangered or protected species were involved in this study.

### Effect of canal-cutting behaviors on laticifers of *Chamaesyce maculata*

*T*. *zethus* larvae were reared from egg hatch within sleeves on mature potted plants of *C*. *maculata*, a widespread prostate plant with narrow stems and small leaves that emit profuse latex when damaged. *C*. *maculata* occurs throughout the continental U.S. including in southern Arizona (http://plants.usda.gov/core/profile?symbol=chma15). To determine how larvae disarm laticifers, ten larvae in the middle or late first, second, third and fifth (final) instars were transferred to new *C*. *maculata* plants not previously used for rearing (one larva/plant). The time each caterpillar spent canal cutting was recorded, then each larva was removed from the plant when it began to feed on a leaf. To quantify the effects of canal cutting on latex exudation, the leaf was severed 5 mm from the tip. Latex flowing from the attached basal portion of the leaf was collected with 2 or 10 μl capillaries (Drummond Microcaps, Broomall, Pennsylvania, USA) and the volume of latex exudate was estimated from the fraction of the capillary filled with latex. As a control, latex outflow was measured in the same way for a previously undamaged leaf that closely matched the fed-upon leaf in size and age, but was located on another stem of the same plant. Which leaf was damaged first for latex collection was determined randomly. For each instar, the volume of latex emitted by control leaves and leaves treated with canal cuts was compared with a Wilcoxon signed rank test. JMP v. 11 was used for all statistical analyses.

### Do *T*. *zethus* larvae disable euphorb laticifers with saliva or VEG secretion?

To test if *T*. *zethus* larvae use saliva or VEG secretions to reduce latex exudation, final instars were tested on poinsettia leaves, which have robust midribs and petioles that cannot be easily crimped with mandibles, unlike the slender stems and petioles of *C*. *maculata*. Three caterpillar treatments were tested: (1) intact caterpillars, (2) larvae with their spinneret cauterized to prevent saliva release from the labial salivary glands, and (3) larvae with the opening to the ventral eversible gland (VEG) sealed to prevent acid secretion from the VEG (N = 8 replicates/treatment).

Larvae were reared on excised poinsettia to the early penultimate instar, then sleeved on mature potted poinsettia plants to gain experience with latex. Young larvae suffer high mortality on intact poinsettia leaves due to copious latex emission, but they survive readily on excised leaves which have depressurized latex canals. Larvae in the early to middle final instar were randomly assigned to treatment. To immobilize larvae, each caterpillar held individually in a vial was submerged in crushed ice for at least 10 minutes. In treatment 2, the spinneret was cauterized with an ART-E1 electrosurgery unit (Bonart Company, Ltd. New Taipei City, Taiwan) using a sharpened fine tip. A micromanipulator was used to position the cautery tip directly on the spinneret viewed at 50x under a dissecting microscope. The Bonart unit allowed more precise cautery than the heated probes and heat pens that have been used previously to ablate spinnerets [29–31]; the spinneret could be readily cauterized without any visible harm to the adjacent sensory pegs. To block the VEG opening for treatment 3, Permatex superglue containing ethyl cyanoacrylate was applied to the opening using a fine pin under the dissecting microscope. Afterwards larvae were placed in the refrigerator for 10 minutes to allow the superglue to harden. All larvae (including the intact controls) were allowed to recover for 5 hours. Only larvae that fed on excised poinsettia leaves during the 5 hour recovery period continued in the experiment. At the end of the 5 hour period, larvae in treatment 3 were examined under a microscope to check that the VEG was still blocked. Each larva was also pinched on filter paper treated with alkaline phenolphthalein to verify that the larva was unable to release acid from the VEG.

Larvae were then weighed and placed individually on the youngest mature leaf on a poinsettia stem. A separate mature potted plant was used with each larva. I recorded when larvae began canal cutting and when they started feeding to determine total time spent pretreating a leaf before feeding. All larvae were removed from the plant when they started to feed. Larvae in treatment 3 were re-examined in the microscope at 50x and retested with phenolphthalein paper; larvae were excluded from the study if their VEG was no longer sealed.

To determine how *Theroa* larvae in the three treatments affect latex outflow, I severed the fed-upon leaf one cm from the tip and collected latex exuding from the leaf with filter paper, which was weighed to determine latex wet weight. Next I cut off an additional 5 cm from the leaf tip and again weighed latex exuding from the leaf. Latex outflow was similarly measured from eight previously undamaged control plants using leaves of similar age and size. The three treatments and control were compared in the weight of latex exuding at 1 cm and at 5 cm from the leaf tip using Kruskal-Wallis tests since the 1 cm data violated the normality assumption of ANOVA. Steel-Dwass tests were used for pairwise posthoc comparisons.

Caterpillars produced conspicuous furrows in leaf midribs. To quantify larval damage to the midribs, leaves were photographed from the side next to a ruler. Image J was used to measure the depth at the center of the deepest furrow for each caterpillar, which was then divided by the depth of the midrib before furrowing (estimated by averaging the depths of the undamaged midrib on each side of the furrow). The three caterpillar treatments were compared using Kruskal-Wallis tests followed by pairwise comparisons with Steel-Dwass tests.

### When do larvae apply VEG secretions?

The ventral eversible glands of final instar *T*. *zethus* larvae were emptied by pinching the larvae repeatedly with fine forceps until they no longer sprayed. Chilled larvae under a dissecting microscope at 50x were then injected in the VEG with up to 10μl of 0.001M toluidine blue dye using a Hamilton 10 μl syringe with a 23s-26s cone tip (Sigma Aldrich, St. Louis, Missouri, USA). After returning to room temperature, larvae readily sprayed blue dye in any direction when pinched, documenting that the VEG was still functional (S2 Movie). To determine when *T*. *zethus* larvae apply VEG secretion to poinsettia midribs, a larva freshly injected with dye was allowed to create a midrib furrow on mature potted poinsettia while being filmed with a Wild M400 photomacroscope outfitted with a Canon T3i camera (S7 movie).

### Do VEG secretions suffice to decrease latex exudation?

To test if VEG secretions alone are sufficient to wither leaf veins and reduce latex exudation, poinsettia midribs were treated with the following four solutions: 5 μl VEG secretion; 5 μl ground labial salivary glands; 5 μl ground labial salivary glands followed 5 minutes later by 5 μl VEG secretion; 5 μl water control (N = 6 replicates/treatment). The third treatment simulates the sequential application of fluids by *T*. *zethus* larvae, which appear to apply saliva while mandibulating the midrib, then VEG acid while pressing the VEG opening against the midrib. The VEG dissected from four final instar *T*. *zethus* contained 14 ± 1 μl (mean ± 1 SEM); thus, larvae have available substantially more VEG secretion than the 5 μl volume selected for this experiment. Wiretrol micropipets (1–5μl, Drummond Scientific, Broomall, Pennsylvania, USA) were used to dispense fluids on the ventral midrib of mature poinsettia leaves held upside down. Fluids were spread over ~1cm of the midrib in the middle of the leaf where the midrib was 2 mm wide. Mature potted plants were randomly assigned to treatment; only one leaf was used on each plant.

VEG secretion was collected from final instars by inserting the caterpillar’s head into a glass vial and repeatedly pinching its body with forceps to stimulate discharge. A total of 661 μl secretion was collected from 85 larvae (7.8 μl/larva). To determine the pH of VEG secretion, spray was collected from three additional sets of 25 final instar *T*. *zethus* larvae. The pH of 100μl from each collection was determined using a micro pH electrode (#5473901, Van London Company, Houston, Texas, USA) and Oaklon 510 pH meter (Vernon Hills, Illinois, USA).

Paired labial salivary glands were dissected from 20 final instars. The elongate tubular glands were blotted to remove hemolymph, then ground in a Biomasher II tissue grinder (Kimble Chase, Vineland, New Jersey, USA).

Twenty four hours after fluids were applied to the midribs, poinsettia leaves were severed 6 cm from the tip (distal to the site of fluid application) and all latex exuding from the attached leaf was collected onto filter paper, which was reweighed to give wet weight of latex exudate. The data violated the normality assumption of ANOVA so the four treatments were compared with a Kruskal-Wallis test followed by pairwise posthoc comparisons using Steel-Dwass tests. Each midrib was then photographed from the side. Image J was used to estimate the extent of withering by measuring the depth of the furrow and adjacent intact midrib as described previously. Kruskal-Wallis and Steel-Dwass tests were again used to compare treatments in midrib depth.

### Are VEG secretions and saliva required for growth on poinsettia?

To determine if *T*. *zethus* larvae require an intact VEG to develop on poinsettia, I compared the growth of intact controls and larvae with a blocked VEG. Larvae in each group were tested on both intact plants and on excised leaves drained of latex (N = 10 replicates/treatment). The goal of the excised leaf treatments was to test if larvae with a blocked VEG can feed as effectively as intact larvae. If so, any differences in growth on intact plants can be attributed to the treatment and not to reduced feeding due to damage caused by blocking the VEG.

Larvae were reared initially on excised poinsettia leaves, then transferred in the early penultimate instar larvae to mature potted poinsettia to gain experience with latex. Larvae in the early final instar were randomly assigned to treatment, then chilled on ice. To block the VEG, a drop of superglue was placed over the VEG opening as described previously. All larvae were allowed to recover five hours with an excised poinsettia leaf provided for food. Larvae that did not feed during the recovery period or that removed the VEG seal were excluded from the study. The remaining larvae were weighed, then offered either an excised mature poinsettia leaf in a plastic box or an intact mature leaf on a potted poinsettia plant ~4 months old. Excised leaves were bled of latex by transecting the petiole three times; each cut was made only after latex outflow from the previous cut ceased. The leaf was then cut in half. Larvae released on plants were free to move, although most stayed on the leaf where they were placed. After 16 hours, each larva was reweighed, then carefully examined to insure the VEG was still blocked. I assessed the effects of caterpillar manipulation and leaf treatment (excised vs. intact leaves) on larval weight change using a two way ANOVA, followed by a one way ANOVA for comparing each leaf treatment separately.

## Results

### Effect of canal-cutting behaviors on laticifers of *Chamaesyce maculata*

**Fig 2:**
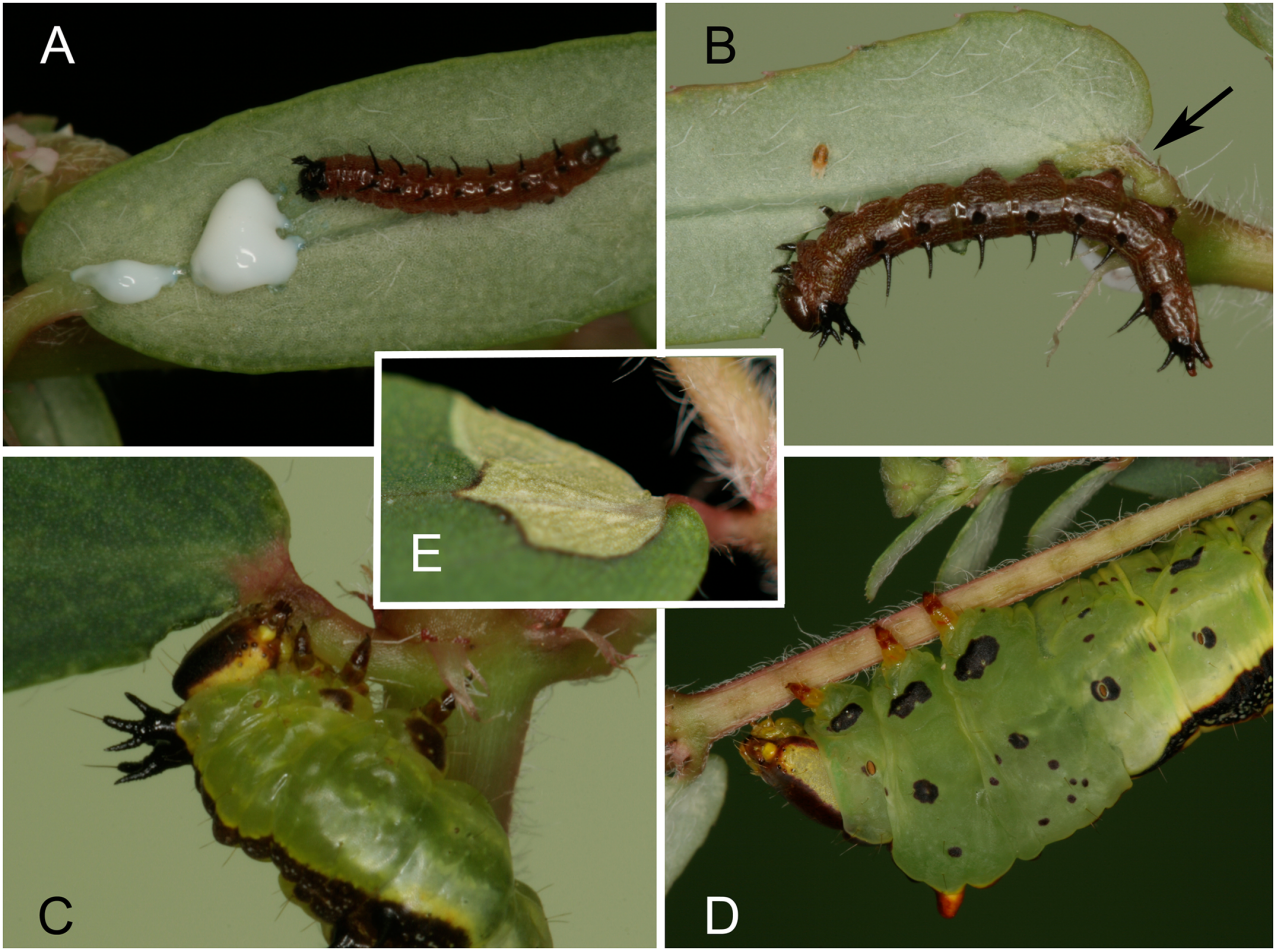
*Theroa zethus* larvae on *Chamaesyce maculata*. (A) First instar disabling latex canals by biting repeatedly into the leaf midrib before feeding distal to the cuts. (B) Second instar feeding after constricting the petiole. (C) Third instar compressing the petiole with its mandibles. (D) Final instar deactivating the laticifers in the stem by repeatedly pinching the stem with its mandibles; ~10 dark spots along the stem indicate the location of the constrictions. (E) Necrotic zone developing in a *C*. *maculata* leaf adjacent to a petiole constriction produced by a second instar *T*. *zethus*.

All 10 first instar caterpillars bit repeatedly into the midrib of a *C*. *maculata* leaf before feeding. Bites almost always progressed from the leaf base towards the tip with the larva backing up and thus keeping clear of the sizable latex drops that emerged (Fig 2A). Second and third instar larvae all used their mandibles to grasp and compress the petiole at the base of the leaf blade or less frequently the leaf midrib (Fig 2B and 2C). Most caterpillars constricted the petioles of multiple leaves before initiating feeding. Leaves with constricted petioles could often be identified visually by a necrotic zone that developed in the leaf blade around the constriction site (Fig 2E).

Seven of the ten final instar larvae used their mandibles to crimp the stem repeatedly before feeding distal to the constrictions (Fig 2D). The larvae alternately reached their head around the stem clockwise and counterclockwise so the stem was pinched from different orientations, presumably to rupture internally the laticifers running along all sides of the stem. Six of the seven also constricted the petiole of at least one leaf before feeding. No latex emission was observed during crimping by any of the second, third or final instar larvae.

**Fig 3:**
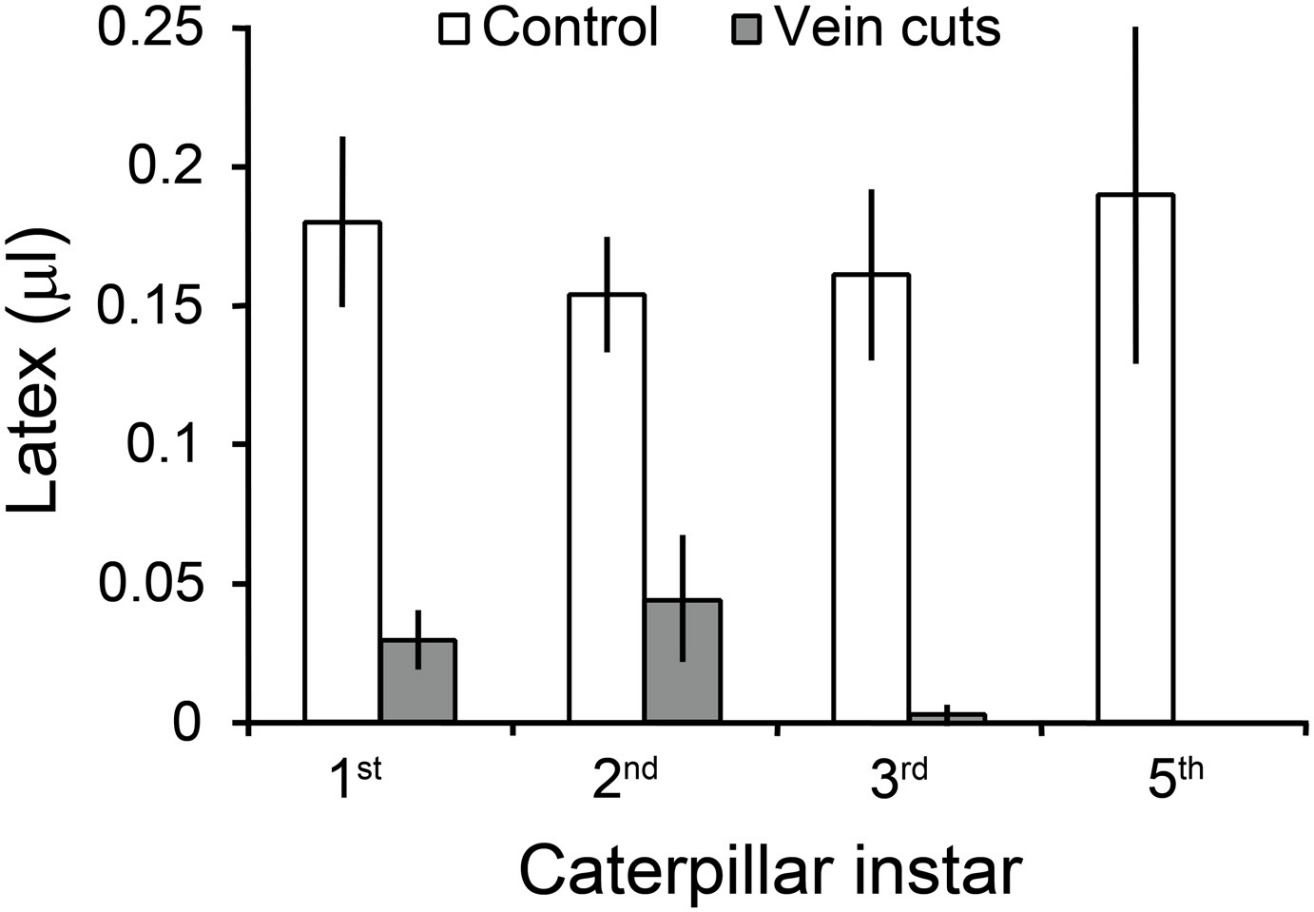
Volume of latex exuding from *C*. *maculata* leaves severed 5 mm from the tip. For each instar, leaves pretreated by *T*. *zethus* larvae with vein cuts or petiole/stem constrictions released significantly less latex than control leaves. N = 10 for each instar, except N = 7 for the final instar; data are presented as means ± 1 SEM.

**Fig 4:**
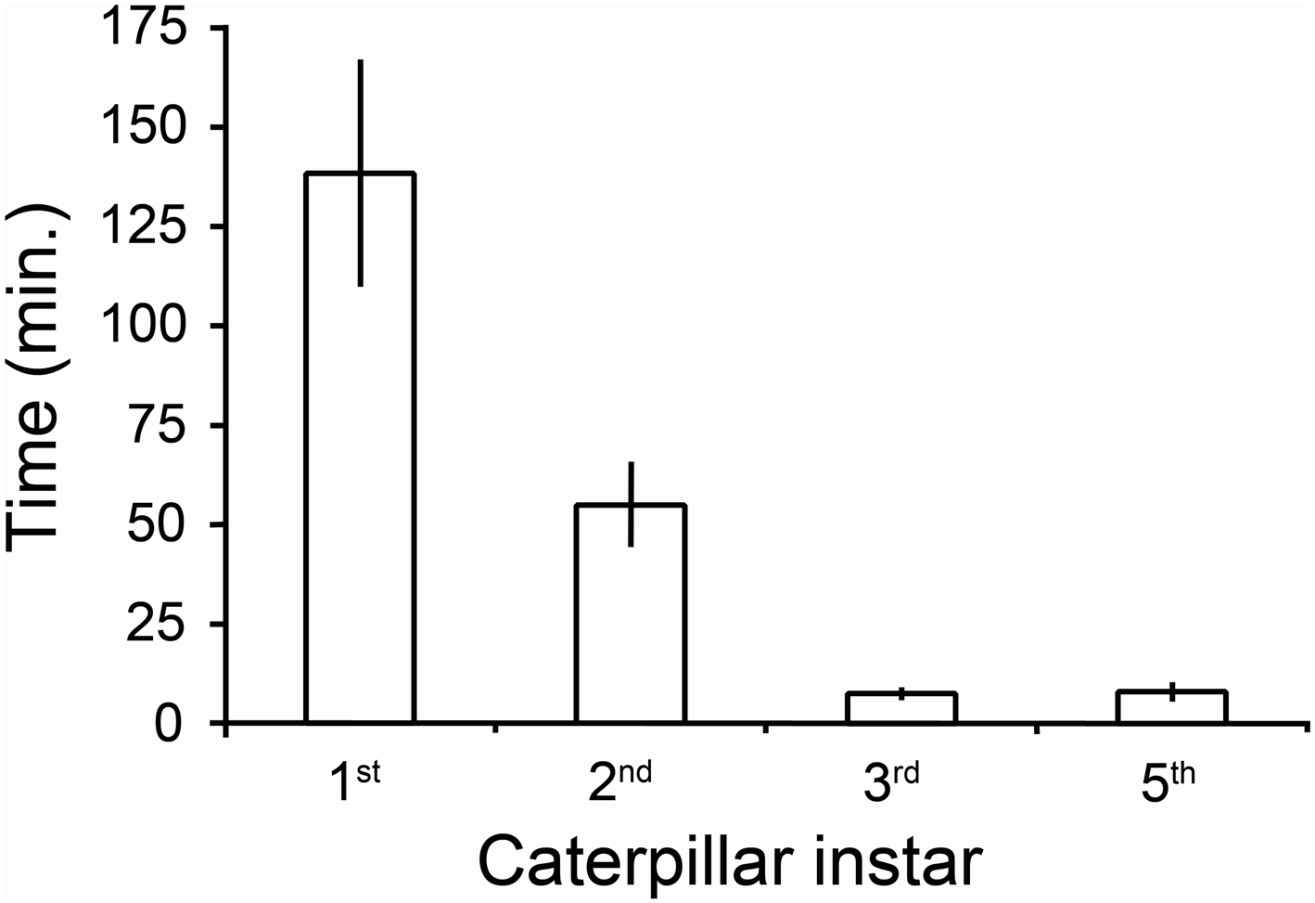
Time *T*. *zethus* larvae spent cutting veins or constricting petioles/stems before initiating feeding. All larvae were tested on *C*. *maculata*. N = 10 for first and third instars, N = 9 for the second instar, N = 7 for the final instar; data are presented as means ±1 SEM.

For each instar tested, midrib cuts and petiole constrictions significantly reduced latex emission distal to the cuts in comparison with control leaves (*P* < 0.01, one tailed Wilcoxon Signed Rank Tests, Fig 3). Larvae initiated feeding on this distal section with reduced exudation. Larger larvae with their more substantial mandibles and musculature more effectively deactivated the latex canals and required less time (Fig 4). First instar larvae on average spent over two hours cutting the midrib of a single leaf before feeding. Second and third instars constricted the midrib or petioles of multiple leaves before initiating feeding and required much less time per leaf (32 ± 8 and 5 ± 1 minutes/leaf respectively, means ± 1 SEM).

### Do *Theroa* larvae disable euphorb laticifers with saliva or VEG secretion?

**Fig 5:**
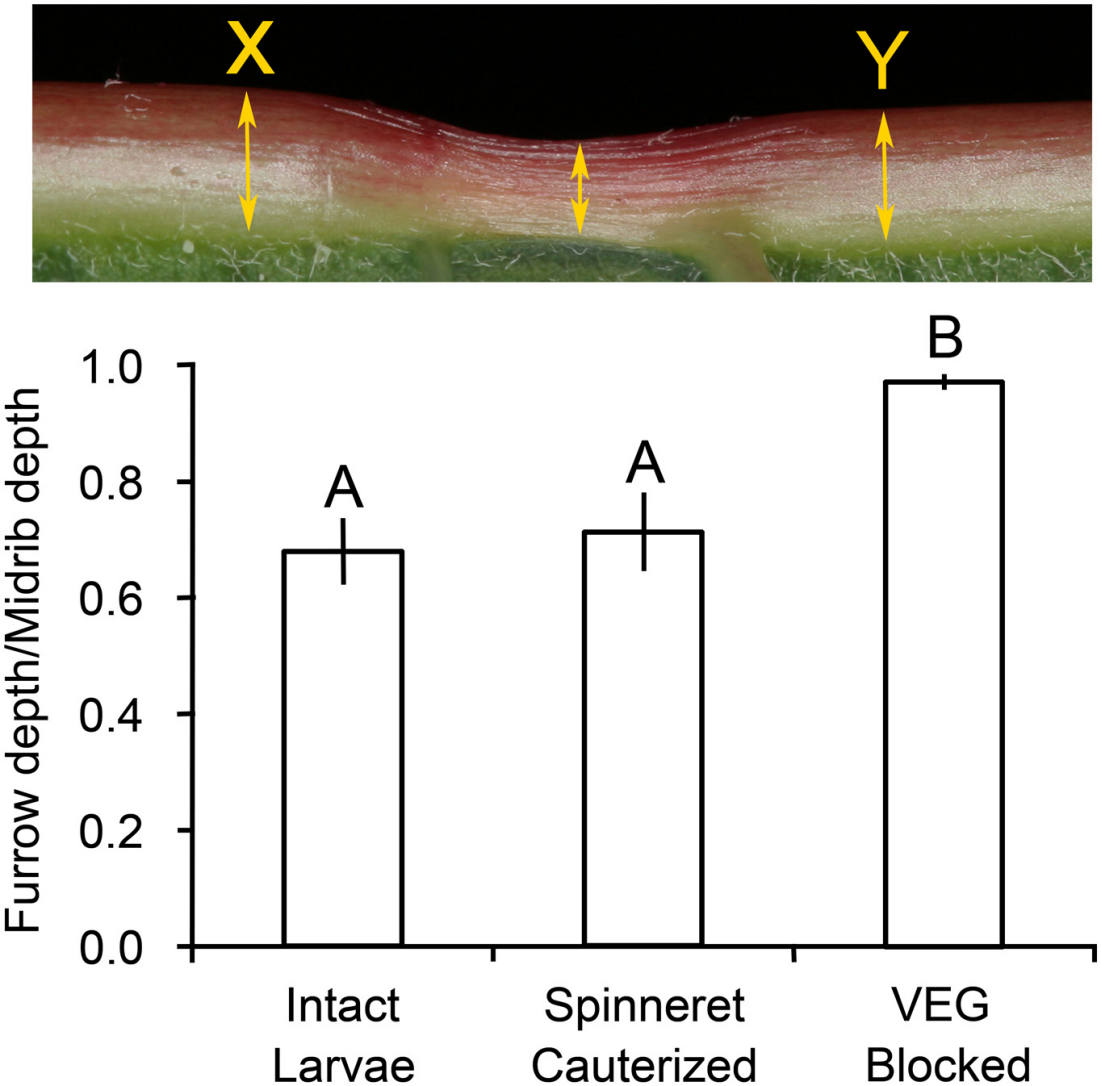
Depth of furrows created by *T*. *zethus* larvae in poinsettia midribs. The depth of the midrib in the center of a furrow was divided by the original midrib depth, which was estimated by averaging depths at X and Y on each side of the furrow. The top photograph shows a furrow created by an intact *T*. *zethus* larva. Caterpillars with a blocked VEG did not decrease midrib depth at all, in contrast to larvae with a functional VEG (intact larvae and larvae with their spinneret blocked). Data are presented as means ±1 SEM; bars with different letters differ significantly at *P* < 0.05 using Steel-Dwass tests.

Larvae with a functional VEG produced conspicuous furrows in leaf midribs (Fig 5). The caterpillars did not cut away leaf tissue with their mandibles; instead, the tissue withered from the application of acid from the VEG. No furrows appeared in leaves attacked by larvae with a blocked VEG or control leaves without caterpillars. Measurement of midrib depth documented that intact larvae and larvae with cauterized spinnerets decreased midrib depth by approximately a third in the furrows, significantly more than the blocked-VEG larvae (*P* < 0.05 Steel-Dwass, Fig 5) which did not alter midrib depth. Slight discoloration of the red poinsettia midribs indicated the location of their attempted furrows.

**Fig 6:**
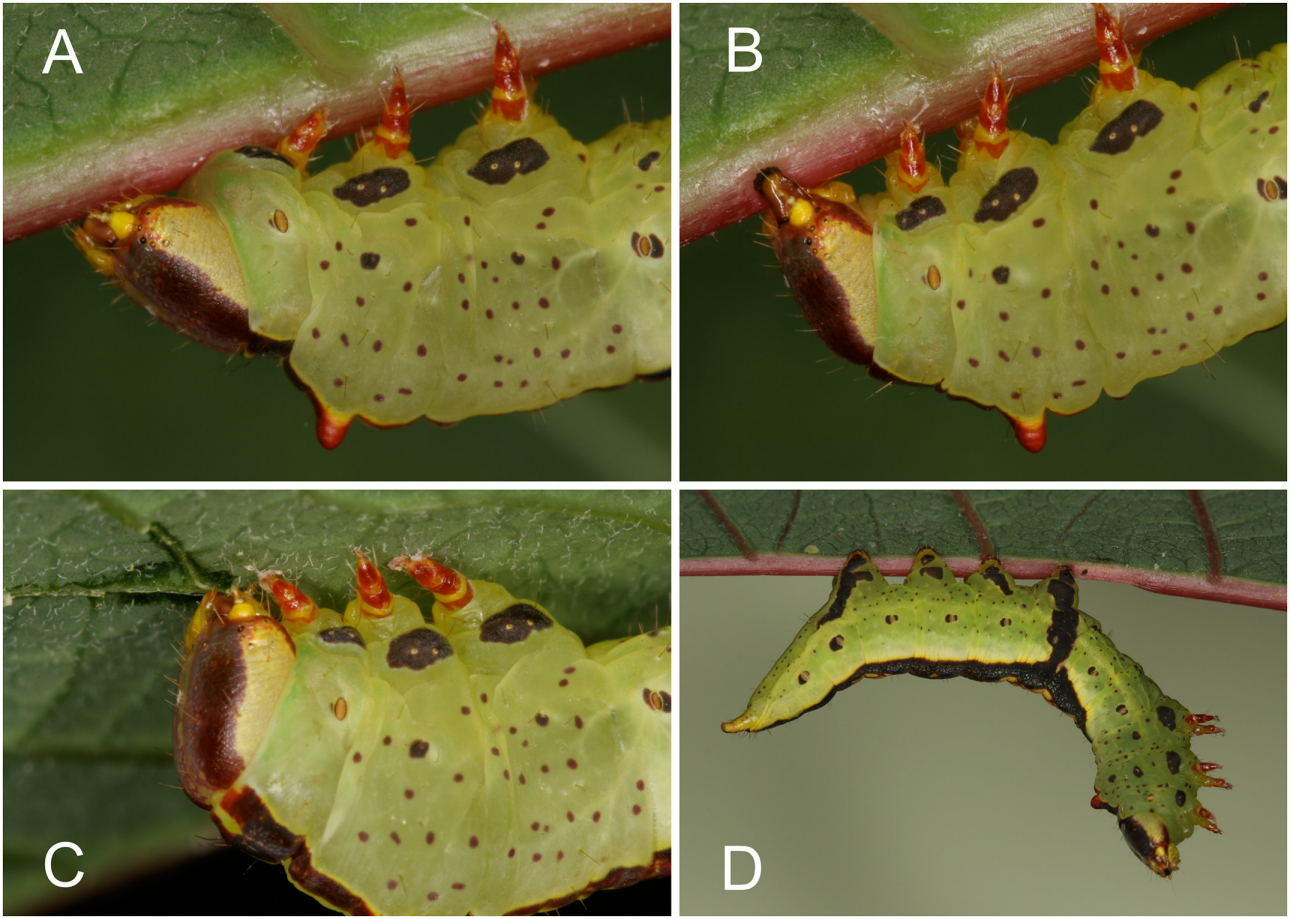
Behaviors of final instar *T*. *zethus* larvae on poinsettia. Control larvae pressing the VEG opening against the midrib surface (A), constricting the midrib (B) and cutting small leaf veins (C). Spinneret-cauterized larva hanging motionless from a leaf (D).

All caterpillars, including those with a disabled VEG, periodically pressed the opening of the VEG against the midrib for approximately 1–3 minutes (Fig 6A; S3 and S4 Movies). They remained motionless during this time except for rhythmic contractions around the VEG. Before and after, larvae actively mandibulated the midrib at the same location, compressing and scraping the midrib (Fig 6B). As acid from the VEG softened the midrib, their mandibles became increasingly effective at compressing tough vascular tissues (S5 Movie). While mandibulating the midrib, larvae repeatedly rubbed their labium over the midrib leaving behind visible fluid (S6 Movie), presumably saliva secreted by the labial salivary glands and emitted through the spinneret. Intact larvae required approximately an hour to complete a single furrow.

**Fig 7:**
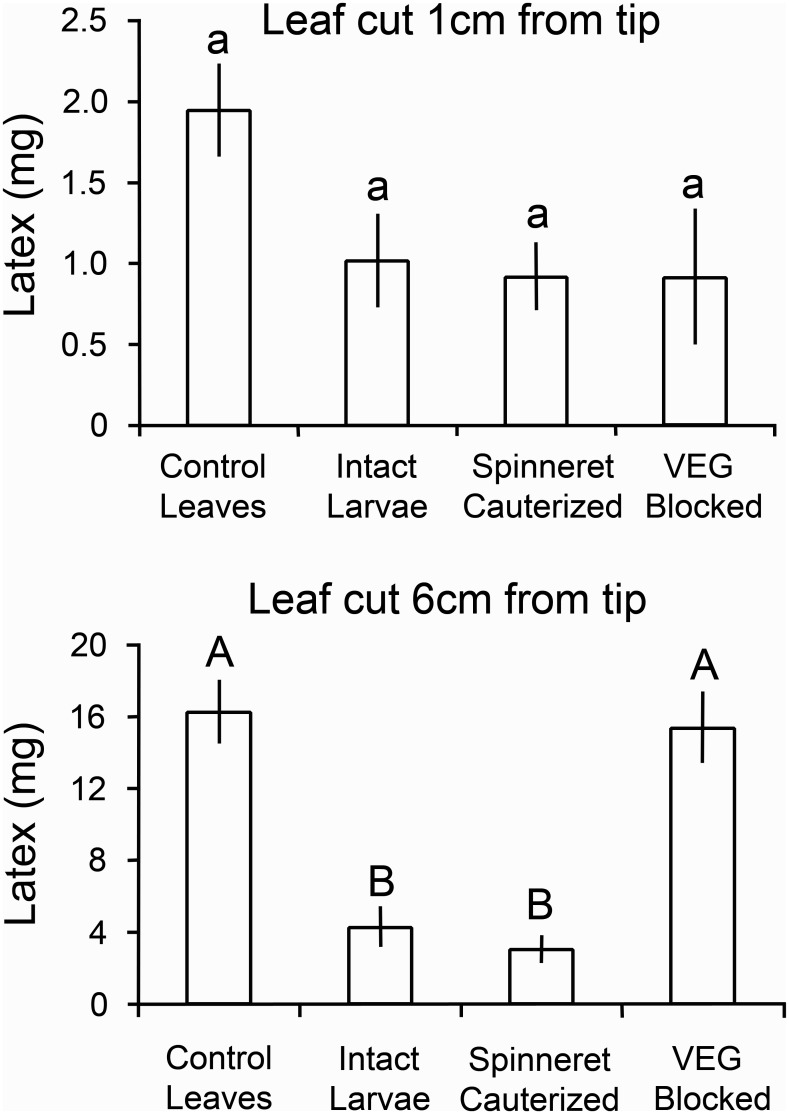
Wet weight of latex exuding from poinsettia leaves. Leaves were cut 1 cm (top) and 6 cm (bottom) from the leaf tip. All larvae were able to compress the midrib near the leaf tip, and thereby reduce latex emission at 1 cm; only larvae with a functional VEG were successful in significantly reducing latex levels 6 cm from the leaf tip. Data are presented as means ± 1 SEM; bars with different letters differ significantly at *P* < 0.05 using Steel-Dwass tests.

In addition to producing furrows in the leaf midrib, larvae tested on poinsettia also often compressed and chewed on small leaf veins near the leaf tip where the veins are sufficiently small to fit within their mandibles (Fig 6C). Larvae invariably initiated feeding near the leaf tip distal to furrows and vein constrictions. Since all of the larvae had functional mandibles, all three caterpillar treatments were able to reduce latex outflow 1 cm from the leaf tip by approximately half relative to control leaves (Fig 7 top). The four treatments differed significantly in latex emission (*P* < 0.05 Kruskal-Wallis), but none of the differences between paired treatments were significant (*P* > 0.05 Steel-Dwass tests) due to substantial variation in latex levels and small sample sizes. However, with cuts made 6 cm from the tip, the intact larvae and the spinneret-cauterized larvae both reduced latex emission significantly in comparison with the control (*P* < 0.005, Steel-Dwass tests; Fig 7 bottom). Caterpillars in both of these treatments had a functional VEG, which was required to depressurize laticifers over the entire end of the leaf. Larvae with a blocked VEG were only able to reduce latex emission at the tip by constricting small veins.

Time until feeding did not differ significantly between the three caterpillar treatments (*P* = 0.15, Kruskal-Wallis test); larvae required an extraordinarily long time ranging from 2.2 to 11.4 hours to disable latex canals and begin feeding (intact larvae 245 ± 32 min., spinneret cauterized 375 ±53 min., VEG blocked 293 ± 61 min.; means ± 1 SEM). Larvae spent most of this time mandibulating the leaf midrib and pressing the VEG opening against the midrib. All larvae with cauterized spinnerets (and infrequently larvae in other treatments) were repeatedly observed hanging from the leaf by their prolegs, motionless but not paralyzed (Fig 6D). The prolonged furrowing and unusual hanging behavior of spinneret-cauterized larvae could be an artifact; cautery creates a wound that may be highly sensitive to acid deposited onto the midrib surface by the VEG.

Additional intact final instar larvae filmed on poinsettia while producing a midrib furrow (5 larvae) or petiole girdle (1 larva) required 39 ± 10 min. to complete a single furrow or girdle. The larvae paused to press their VEG against the midrib or petiole 8 ± 2 times, each lasting 134 ± 14 sec (means ±1 SEM, N = 48). Overall, the six larvae spent 50 ± 7% of their time immobile with the VEG held tightly against the midrib or petiole. The remainder of the time was mostly devoted to mandibulating the midrib/petiole and rubbing the labium over its surface.

### When do larvae apply VEG secretions?

No blue dye from the VEG was released when larvae mandibulated the midrib or rubbed their labium over the midrib surface (S7 Movie). The VEG opening was not visible while larvae were holding it pressed against the midrib, and thus it was not possible to see directly if dye was released. However, when a larva lifted its thorax off the midrib, blue fluid was clearly visible, documenting that the VEG secretes fluid while larvae press their VEG opening against the midrib (S7 Movie). Pulsating thoracic contractions around the VEG presumably function to pulse acid out (perhaps in and out) of the VEG (S4 Movie). Since larvae press their prothorax against the midrib, secreted fluid is usually localized under the larva.

### Do VEG secretions suffice to decrease latex exudation?

**Fig 8:**
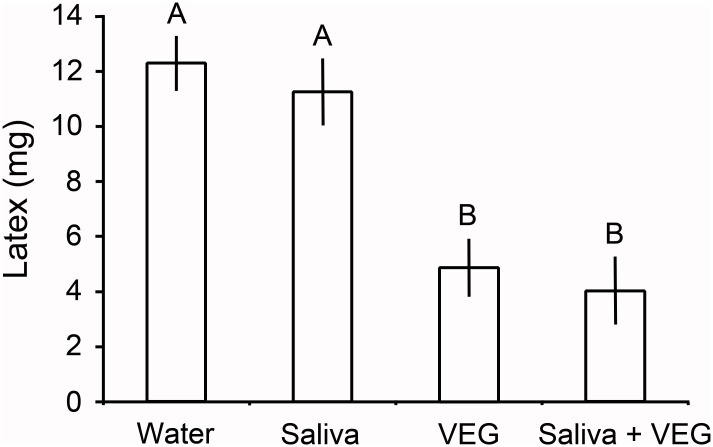
Wet weight of latex exuding from poinsettia leaves. Leaves were cut 6 cm from the leaf tip after secretions were applied to the leaf midrib 24 hours previously. VEG secretions reduced latex exudation whether tested alone or with saliva (ground salivary glands). Data are presented as means± 1 SEM; bars with different letters differ significantly (*P* < 0.05 Steel-Dwass tests).

Poinsettia leaves treated with larval secretions differed in the volume of latex released (*P* < 0.001 Kruskal-Wallis, Fig 8). VEG secretion by itself sufficed to create a pronounced furrow in the poinsettia leaf midribs and to reduce distal exudation by 60%. The VEG secretion proved to be highly acidic (pH = 0.55 ± 0.06; mean ±1 SEM). Leaves treated with VEG secretion did not differ in latex release compared to leaves treated with saliva (ground salivary glands) followed by VEG secretion (*P* = 0.9 Steel-Dwass). However, significantly less latex was released from leaves treated with VEG secretion or with saliva + VEG secretion compared to the water control (*P* < 0.05 Steel-Dwass tests). Leaves treated with saliva alone did not differ from the water control (*P* = 0.9 Steel-Dwass).

**Fig 9:**
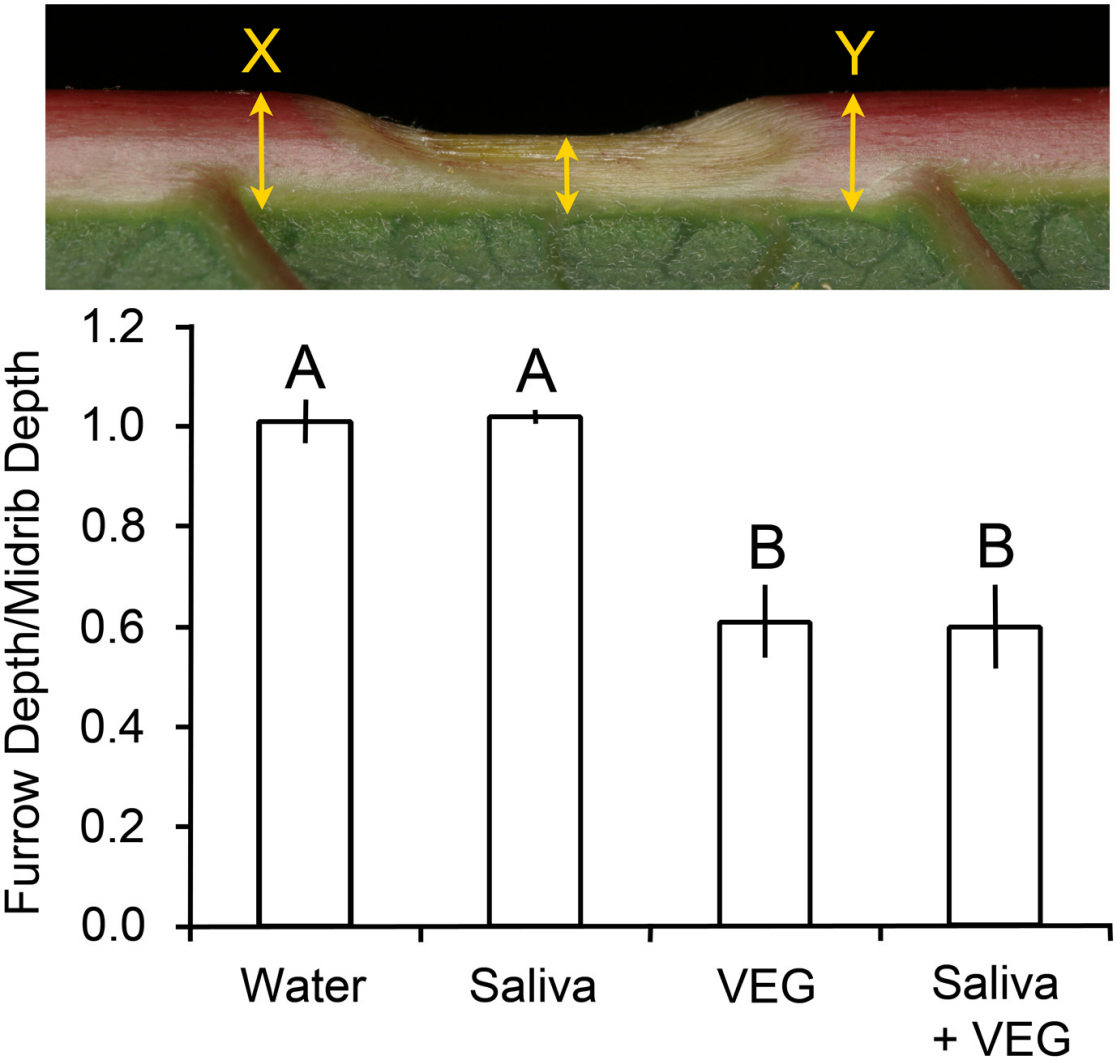
Depth of furrows created in poinsettia midribs by *T*. *zethus* secretions. The depth in the center of a furrow was divided by the original midrib depth, which was estimated by averaging depths at X and Y on each side of the furrow. Only the VEG secretion alone or together with ground salivary glands produced a detectable furrow. Data are presented as means ± 1 SEM; bars with different letters differ significantly (*P* < 0.05 Steel-Dwass tests). The photograph at the top shows a furrow created by 5 μl of VEG secretion, which resembles furrows created by intact *T*. *zethus* larvae as illustrated in Fig 5.

Only midribs treated with VEG secretion alone or saliva + VEG secretion showed detectable withering (Fig 9). Both VEG treatments decreased midrib depth by 39%, significantly more than the water control (*P* < 0.05, Steel-Dwass tests). Ground salivary glands applied to leaf midribs did not decrease midrib depth relative to the water control (*P* = 1.0 Steel-Dwass, Fig 9), nor did the saliva alter the depth of furrows in the saliva + VEG secretion relative to the VEG alone treatment (*P* = 1.0).

### Are VEG secretions and saliva required for growth on poinsettia?

**Fig 10:**
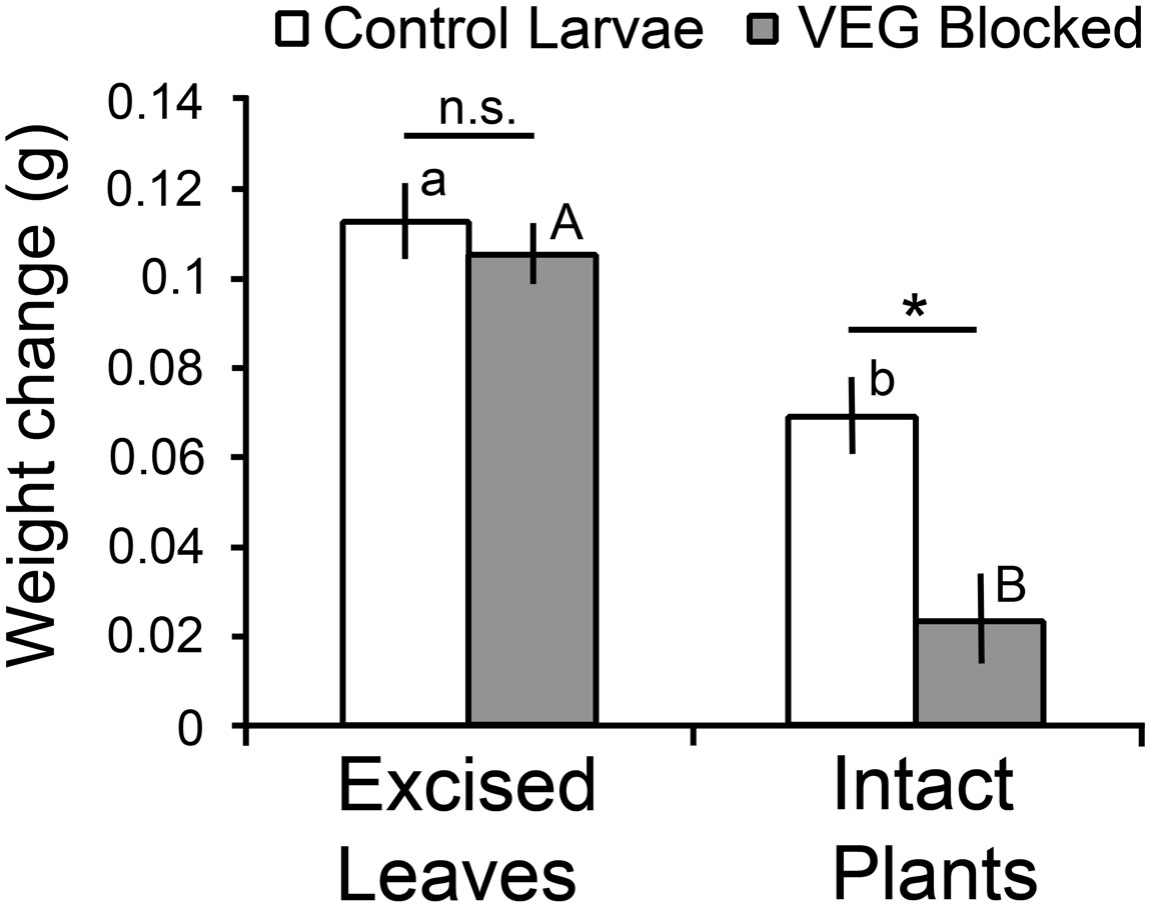
Growth of final instar larvae of *T*. *zethus* on poinsettia over 16 hours. Control and VEG-blocked larvae differed significantly in weight change on intact plants (* *P* < 0.005, one way ANOVA), but not on excised leaves (n.s. = not significant). Larvae grew more slowly on the intact plants, especially when their VEG opening was blocked. Treatments that differed significantly (*P* < 0.001, *t*-tests) have different letters above the bars (lowercase letters comparing control larvae, upper case comparing VEG-blocked larvae). Data are presented as means ±1 SEM; N = 10 larvae/treatment.

Caterpillar manipulation, leaf treatment, and their interaction all significantly affected larval weight change (two way ANOVA: caterpillar F_1,36_ = 10.2, *P* < 0.005; leaf F_1,36_ = 58.5, *P* < 0.0001; interaction F_1,36_ = 5.4, *P* < 0.05)(Fig 10). On excised leaves, caterpillar treatment did not affect larval weight change (one way ANOVA, F_1,18_ = 0.5, *P* = 0.51) documenting that VEG blockage did not interfere with the caterpillars’ ability to feed. However, on intact plants, VEG-blocked larvae gained significantly less weight than control larvae (one way ANOVA, F_1,18_ = 12.8, *P* < 0.005). Larvae unable to release acid from the VEG on average gained only 34% as much weight on intact plants as the control larvae. Furthermore, the control larvae on intact plants gained only 61% as much weight as the controls on excised leaves, a significant difference (*P* < 0.001, *t*-test). This reduction in growth on intact plants was undoubtedly due to the laborious and time-consuming task of deactivating the laticifer system. On intact plants, larvae fed distal to furrows and vein constrictions, whereas larvae on excised leaves only rarely produced a furrow or constricted veins and then fed on either the base or tip of the leaf.

### Furrows and girdles on hostplants in the field

**Fig 11:**
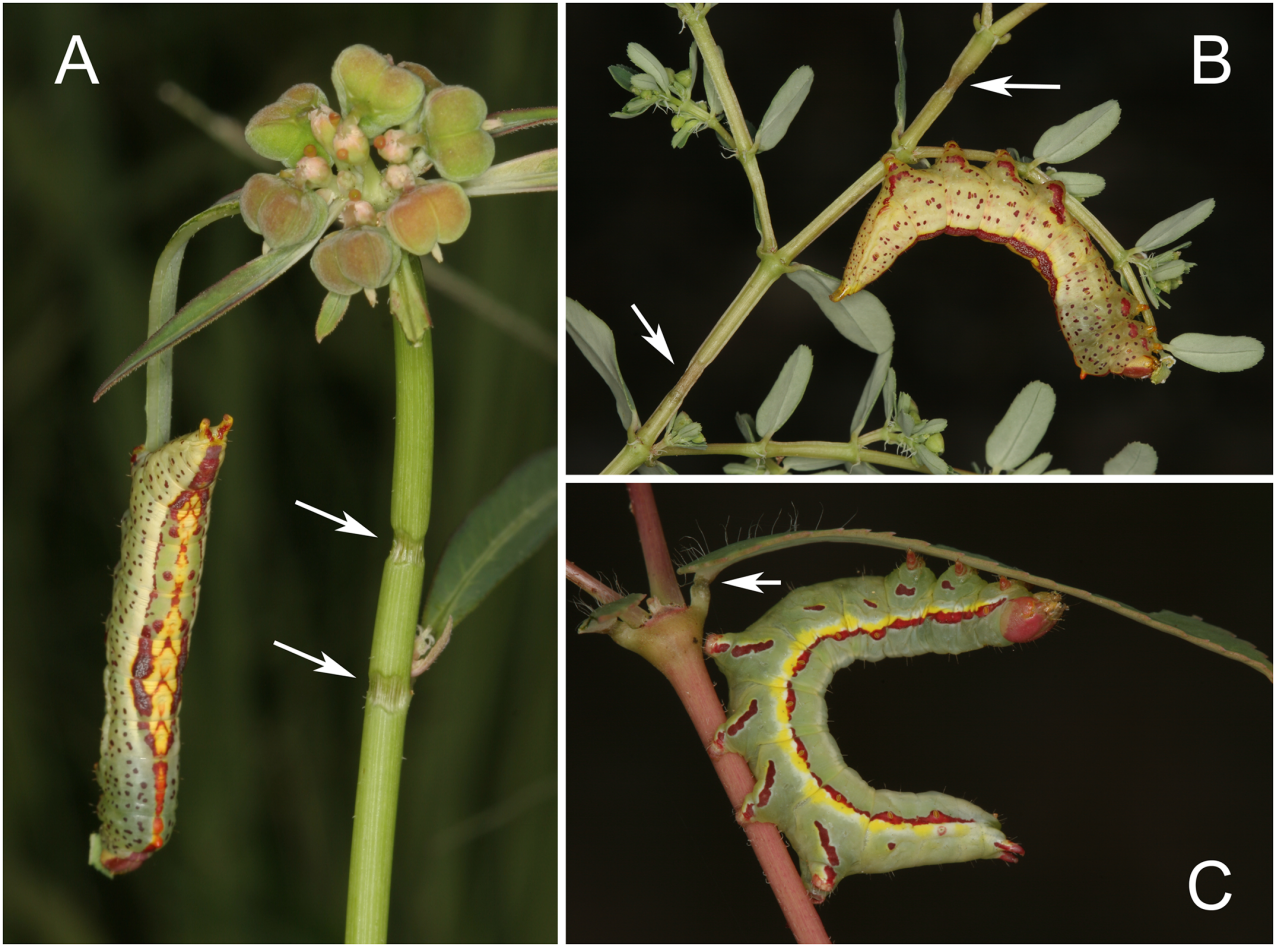
Final instar *T*. *zethus* and *Praeschausia zapata* photographed in the field in Arizona. *T*. *zethus* feeding beyond girdles in (A) *Euphorbia cyathophora*, and (B) *Chamaesyce serpyllifolia*. (C) *P*. *zapata* feeding on *C*. *hyssopifolia* after constricting the petiole.

To determine if the behavior of *T*. *zethus* larvae on *C*. *maculata* and *E*. *pulcherrima* resembles the behavior of larvae on natural hostplants, *T*. *zethus* larvae were observed in the field in southern Arizona during August 17–20, 2012. The larvae occurred in abundance on an upright euphorb *Chamaesyce hyssopifolia*, and were also found on *Euphorbia cyathophora*, *E*. *dentata*, and a prostrate euphorb, *C*. *serpyllifolia*, that resembles *C*. *maculata* in morphology. On all four foodplants, the larvae always fed distal to withered girdles in stems or petioles (Fig 11A and 11B). Observations of late instar larvae on *C*. *hyssopifolia* and *E*. *cyathophora* in the field and on both species grown in the greenhouse confirmed that larvae produced girdles, not by cutting the plant, but by pressing their VEG opening against the plant surface and by mandibulating at the same location, thereby producing a girdle that encircled stems or petioles or creating a furrow in leaf midribs. Thus, the behavior of larvae on natural hostplants closely matched the behavior of larvae observed on *C*. *maculata* and *E*. *pulcherrima* in the lab.

A second notodontid species, *Praeschausia zapata*, which is closely related to *Theroa* (D. Wagner pers. comm.), was also found feeding on the same *Chamaesyce hyssopifolia* plants as *T*. *zethus*. Like *T*. *zethus*, *P*. *zapata* larvae used their mandibles to constrict *C*. *hyssopifolia* petioles and stems, then fed distal to the constrictions (Fig 11C). Whether they also apply acid from the VEG is not known.

## Discussion

*Theroa zethus* is the first notodontid caterpillar reported to employ vein cutting and petiole constriction—behaviors widely used by insect folivores on plants with latex canals or resin ducts. First instar *T*. *zethus* on *C*. *maculata* repeatedly bit into the midrib producing a series of latex-covered incisions that resembled vein cuts by herbivores on Anacardiaceae, Apocynaceae, Burseraceae, Euphorbiaceae, and Moraceae [10, 13, 17, 32]. Larger *T*. *zethus* larvae on *Chamaesyce* and *Euphorbia* used their mandibles to compress petioles and stems. A few herbivores on latex plants, notably sphingid and danaine caterpillars, have been previously reported to pinch leaf veins, petioles, and flower stalks [10, 13, 16, 17].

The latex system of *Chamaesyce* and *Euphorbia* is highly vulnerable to localized disruption. The non-articulated laticifer cells originate as a small number of cells in the embryo (12 or 24 in *E*. *marginata*)[33]; the cells elongate as the plant grows and form a branching system that extends throughout the stems and leaves [34–36]. Latex is stored under pressure within these living cells. Damage to a leaf vein causes immediate release of latex as laticifer contents flow from high to low pressure. Eventually the rupture seals and latex pressures are restored proximal to the breach. Latex pressures distal to the cuts remain diminished [13, 14, 37, 38]. By cutting a leaf vein with its mandibles, a first instar *T*. *zethus* severs some of the canals and drains some latex from the surrounding area. Subsequent cuts in the vein are made distal to previous cuts, thus progressively depressurizing the distal branches of the laticifers and further isolating this section from the latex reservoir within the elongate laticifer cells.

Constricting leaf veins has a similar effect on the laticifers as vein cuts [13]. Exudation is substantially reduced distal, but not proximal to the constriction. Presumably by pinching veins, the caterpillars rupture and drain laticifers internally. Since the latex only rarely reaches the surface, the larvae effectively disable the laticifer defense without contacting exudate.

Constricting tough vascular tissues in leaf veins requires considerable effort. By applying acid from their anti-predator VEG, *Theroa* softens the veins. VEG secretion alone sufficed to wither poinsettia midribs 24 hours after application and to reduce distal outflow of latex 6 cm from the tip by 60% (Figs 8 and 9). Intact *T*. *zethus* larvae similarly created furrows in poinsettia midribs that reduced exudation by 73% measured 6 cm from the tip (Fig 7). With one or more furrows, the larvae achieved this greater reduction in a shorter time period (4.1 hours on average). VEG secretion by itself typically caused little or no withering in just four hours. *Theroa* larvae more rapidly disabled laticifers by scraping and compressing the midrib extensively with their mandibles and by applying saliva (S5 and S6 Movies). Scraping the midrib may function to remove the waxy surface layer, thus allowing more effective acid penetration. Due to their cuticle coating, epidermal cells are relatively impermeable to protons [39]. Interestingly, plant physiologists studying the effects of acid on plant growth similarly abrade stem surfaces to facilitate acid penetration [39]. Formicine ants, which spray formic acid on enemies, likewise bite where they spray, thus creating routes for the water–soluble acid to penetrate the lipid-coated cuticle of their enemies [40]. *Theroa* larvae able to secrete VEG acid grew significantly faster on intact plants than larvae with a blocked VEG (Fig 10). The ability to create furrows, and thereby disable the laticifer system over a large portion of the leaf, resulted in a threefold increase in growth.

The pH of the apoplast in girdles and furrows is not known, but presumably a gradient is established with VEG acid gradually penetrating into the vein interior with repeated rounds of mandibulation and acid application facilitating penetration. Plant cells exposed to highly acidic conditions suffer membrane damage and loss of turgidity [41], which would explain the withering observed in *T*. *zethus* girdles and furrows. Acid treatment also degrades hemicellulose, thus weakening cell walls [42]. VEG acid may serve not just to degrade cell walls, but also to activate cell wall loosening, particularly in growing cells. Expanding plant cells excrete protons into the apoplast, which activates proteins (expansins) and enzymes that loosen cell walls, thus permitting the cells to increase in size (the acid growth hypothesis)[41, 43]. Notably, the extensibility of cell walls in oat and maize coleoptile sections reached a maximum at the lowest pH values tested (pH 2)[44, 45]. By secreting acid, T. *zethus* may co-opt the plant’s natural acid-growth mechanism for weakening cell walls. Histological examination of cross sections through *Theroa* furrows in poinsettia document that the cell walls remain intact, but are highly distorted [46]. Weakening the walls allows larvae to effectively compress leaf veins with their mandibles (S5 Movie). The constrictions presumably rupture latex canals internally, although VEG secretions tested alone sufficed to reduce distal latex outflow (Fig 9). Apparently VEG acid functions not just to soften tough tissues, but also to directly damage laticifers, perhaps by weakening the walls of these high pressure cells resulting in rupture.

*Theroa* larvae employ a unique behavior when applying acid. The VEG opening located on the underside of the first thoracic segment is pressed tightly against the midrib surface. The larvae remain motionless for several minutes except for rhythmic contractions visible on each side of the VEG opening (S4 Movie). The injection of blue dye into the VEG documented that larvae secrete acid onto the vein surface at this time (S7 Movie). This technique for filling the VEG with blue dye (or other fluids) will facilitate future studies on how the VEG disables host defenses and deters predators.

In addition to the VEG, caterpillars emit secretions from a variety of glands [47], some of which function in defense against predators and parasitoids [48, 49]. To my knowledge, *T*. *zethus* is the first caterpillar reported to use an anti-predator gland for disarming hostplant defenses. Acid secretions are widely employed by other insects in defense and offense. Formicine ants and other insects discharge formic acid to repel enemies [50]. The tropical ant, *Myrmelachista schumanni*, which lives in domatia on plant hosts, kills non-hosts by injecting formic acid into their leaves. The ants thereby create monospecific stands known locally as Devil’s gardens [51]. Ants also use formic acid as an alarm pheromone [52], to detoxify the venom of enemies [53], and to disinfect fungus-exposed brood [54].

Besides applying VEG acid to furrows, *T*. *zethus* caterpillars secrete fluid, presumably saliva from the labial salivary glands, while rubbing the labium over the vein surface (S6 Movie). Larvae wipe the labium over the midrib, not just after encountering latex exudate, but repeatedly while mandibulating the midrib. The role of this secreted fluid is unclear especially since VEG acid applied at the same location presumably disables many salivary enzymes.

## Conclusions

The behaviors of *T*. *zethus* larvae only superficially resemble the girdling of other notodontids such as *Oedemasia leptinoides*. *Oedemasia* larvae create girdles and furrows by laboriously chewing into woody tissues with their mandibles. Similar behavior on euphorbs would cause a continuous release of latex exudate. The vein cutting and vein constricting behaviors of *T*. *zethus* more closely resemble the behaviors of other insects on latex plants, a clear case of convergent evolution. However, *Theroa*’s behaviors also differ. The larvae appear to apply copious amounts of saliva onto the plant surface like *Oedemasia* and they exploit their notodontid weaponry. By softening veins with acid from the VEG, they can constrict and disarm laticifers, often without contacting latex.

## Supporting Information

S1 Data Canal cutting by *T*. *zethus* on *C*. *maculata*. Different instars are compared in time spent cutting/constricting canals and in latex emission beyond the cuts.
(XLSX)

S2 Data Effect of blocking the VEG and the spinneret on depth of furrows produced in poinsettia. (XLSX)

S3 Data Effect of blocking the VEG and the spinneret on latex exudation and time until feeding. (XLSX)

S4 Data Effect of VEG secretion and saliva on latex exudation and furrow depth. (XLSX)

S5 Data Effect of blocking the VEG on growth of *T*. *zethus* larvae on poinsettia. (XLSX)

S1 Movie Final instar *T*. *zethus* discharging its VEG on filter paper impregnated with alkaline phenolphthalein. A simulated attack with forceps causes the larva to repeatedly spray acid from its VEG, thus changing the pH sensitive paper from pink to white.
(MP4)

S2 Movie Final instar *T*. *zethus* larva discharging its VEG contents onto white paper. The spray is light blue because the VEG had been injected with dilute toluidine dye.
(MP4)

S3 Movie Final instar *T*. *zethus* larva creating a furrow in a poinsettia midrib. The larva alternates between mandibulating the midrib and applying acid with the VEG during which it is motionless except for periodic contractions around the VEG. Film speed has been increased 12x.
(MP4)

S4 Movie Final instar *T*. *zethus* larva applying VEG acid to a poinsettia midrib. The larva pulses with rhythmic contractions while holding its VEG opening pressed against a poinsettia midrib.
(MP4)

S5 Movie Final instar *T*. *zethus* using its mandibles to compress a poinsettia petiole. Mandibular contractions are ineffective at compressing hard vascular tissues. However, 41 minutes later, after the larva has repeatedly applied VEG acid and further mandibulated the petiole, the vascular tissues are now soft and easily compressed.
(MP4)

S6 Movie Final instar *T*. *zethus* larva with a blocked VEG bathing the midrib with fluid. The larva repeatedly rubs its labium over the surface while creating a furrow. The fluid covering the surface is presumably saliva secreted by the labial salivary glands and emitted from the spinneret.
(MP4)

S7 Movie Final instar *T*. *zethus* with dilute toluidine dye injected in the VEG. The larva is creating a furrow in a poinsettia midrib. No blue dye appears while the larva mandibulates the midrib or wipes its labium over the surface. However, a blue drop is clearly visible when the larva lifts its thorax after pressing its VEG opening against the midrib for several minutes. The dye documents that VEG contents are released onto the midrib while the immobile larva pulses its prothorax with the VEG tightly held against the surface.
(MP4)
